# Gut Microbiota and Nonalcoholic Fatty Liver Disease: Insights on Mechanism and Application of Metabolomics

**DOI:** 10.3390/ijms17030300

**Published:** 2016-03-15

**Authors:** Xuyun He, Guang Ji, Wei Jia, Houkai Li

**Affiliations:** 1 Center for Chinese Medical Therapy and Systems Biology, Shanghai University of Traditional Chinese Medicine, Shanghai 201203, China; hexuyun1993@126.com; 2 Institute of Digestive Disease, Longhua Hospital, Shanghai University of Traditional Chinese Medicine, Shanghai 201203, China; jiliver@vip.sina.com; 3 Center for Translational Medicine, and Shanghai Key Laboratory of Diabetes Mellitus, Department of Endocrinology and Metabolism, Shanghai Jiao Tong University Affiliated Sixth People’s Hospital, Shanghai 200233, China

**Keywords:** gut microbiota, NAFLD, obesity, insulin resistance, bile acids, choline

## Abstract

Gut microbiota are intricately involved in the development of obesity-related metabolic diseases such as nonalcoholic fatty liver disease (NAFLD), type 2 diabetes, and insulin resistance. In the current review, we discuss the role of gut microbiota in the development of NAFLD by focusing on the mechanisms of gut microbiota-mediated host energy metabolism, insulin resistance, regulation of bile acids and choline metabolism, as well as gut microbiota-targeted therapy. We also discuss the application of a metabolomic approach to characterize gut microbial metabotypes in NAFLD.

## 1. Introduction

Gut microbiota are involved in various aspects of host physiology such as modulation of immunity development, inflammation, and energy metabolism [1]. In 1921, Hoefert *et al.* observed that the structure of intestinal bacteria was significantly altered in patients with chronic liver disease [2]. Moreover, small intestine bacterial overgrowth (SIBO) is directly correlated with the pathological degree in cirrhosis patients [3]. Currently, the dysregulation of gut microbiota is found to be involved in a variety of metabolic diseases such as nonalcoholic fatty liver disease (NAFLD) [4], nonalcoholic steatohepatitis (NASH) [5], diabetes [6,7], insulin resistance [8,9] and obesity [9,10,11].

NAFLD is a complicated metabolic disease with profound interactions between genetic and environmental factors [12]. Studies have indicated that the abundance of *Bacteroidetes* in NASH patients is much lower than that in simple steatosis and healthy groups [13]. In a recent study, germ-free C57BL/6J mice that received intestinal bacteria from high blood glucose mice induced by a high-fat diet, were likely to develop steatosis and insulin resistance compared to counterparts that were transplanted with bacteria from mice with normal blood glucose levels [14]. The data provides direct evidence that gut microbiota is a causative factor for the development of NAFLD and that the gut microbiota-mediated metabolic phenotype is transmissible. It has been recognized that gut microbiota are probably involved in modulating all risk factors for NAFLD formation, such as dysregulation of energy homeostasis, insulin resistance, inflammation, and choline and bile acid metabolism. Metabolomics is a relatively new member of the systems biology family following genomics, transcriptomics and proteomics, which is used to analyze metabolite variations in different conditions such as diseases, drug therapy or genetic modification. The application of metabolomics in NAFLD not only characterizes the disease-related metabolic signatures, but also reveals the involvement of gut microbiota by identifying gut microbial metabolite variations. In this review, we discuss the gut microbial mechanisms of NAFLD in the above-mentioned aspects, as well as the application of a metabolomic approach in the study of NAFLD. 

## 2. The Gut Microbial Mechanism of NAFLD

### 2.1. Gut Microbiota and Host Energy Metabolism

Obesity is the basis of most metabolic diseases. Gut microbiota play important roles in modulating host energy balance and contribute to the development of obesity and obesity-based metabolic diseases [15,16]. Conventional mice usually acquire more body weight than their germ-free litters even on an identical diet, and the body fat of germ-free mice that are transplanted with bacteria from conventional mice is 60% higher than in controls. Gut microbiota promotes the absorption of monosaccharides by the small intestinal mucosa, which accelerates the *de novo* synthesis of fatty acids and suppresses fasting-induced adipocyte factor (FIAF) in intestinal cells, resulting in the accumulation of triglycerides in adipocytes and the development of obesity [17]. 

In the mammalian gastrointestinal tract, over 90% of the microbes are *Firmicutes* and *Bacteroidetes* bacteria [18]. The variation in abundance of *Firmicutes* and *Bacteroidetes* bacteria predisposes to differences in energy metabolism among individuals [15]. In *ob*/*ob* mice, the content of *Bacteroidetes* is half that of normal mice, while the abundance of *Firmicutes* is significantly higher. The transplantation of intestinal bacteria from genetically obese mice to germ-free mice will greatly improve the efficiency of intestinal energy absorption and increase their body weight [19]. Similar results are observed in obese subjects who have a higher abundance of *Firmicutes* and lower numbers of *Bacteroidetes* than their counterparts with normal body weight [19]. Moreover, energy restriction in obese subjects not only reduces their body weight, but also restores the composition of *Firmicutes* and *Bacteroidetes* bacteria in the gastrointestinal tract [15]. In another cohort study, scientists analyzed the composition of gut microbiota in a group of babies aged 6–12 months, and tracked their body weight at seven years of age. It was reported that children of normal body weight usually have a higher percentage of intestinal *Bifidobacterium* and a lower percentage of *Staphylococcus aureus* during infancy, suggesting that differences in gut microbiota occur prior to the occurrence of body weight variation [20]. Therefore, the composition of gut microbiota plays a decisive role in intestinal energy absorption and accordingly influences body weight gain. 

In addition to the signaling modulation, the gut microbial contribution to the host energy harvest is also associated with the production of microbial metabolites. For example, short chain fatty acids (SCFAs) are derived from gut microbiota metabolism including acetate, propionate, butyrate and so on. SCFAs can boost the host energy harvest in the intestinal tract, thus contributing to the development of obesity and obesity-related metabolic diseases [21,22]. Moreover, SCFAs have intestinotrophic effects by stimulating secretion of glucagon-like peptide-2 (GLP-2) in the ileum [23,24], and reducing the gut permeability, which lowers the levels of plasma lipopolysaccharide and inflammatory cytokines [25]. It has been demonstrated that administration of probiotic VSL#3 produces beneficial metabolic effects in obese mice by stimulating butyrate production and the subsequent induction of GLP-1 release from intestinal L-cells [26]. Although lots of exciting experimental evidence has been acquired, the roles and mechanisms of gut microbiota in host energy metabolism still needs further investigation because of the complicated relationship between gut microbiota and host metabolism. 

### 2.2. Gut Microbiota and Insulin Sensitivity

Insulin resistance is a basic pathophysiology of NAFLD, which enhances fat accumulation and initiates inflammatory reactions in hepatocytes [27]. The formation of insulin resistance is associated with dysbiosis [9], whereas probiotic or TNF-α antibody can reduce the release of inflammatory cytokines and inhibit SIBO, leading to an improvement in insulin sensitivity [28,29,30,31]. The exposure to high-dose antibiotics results in alteration of gut microbiota and various beneficial metabolic consequences such as improvement of insulin resistance and glucose tolerance, reduction in body weight and inflammation in both dietary [32] and genetically obese mice [33,34]. However, the metabolic consequences of gut microbial remodeling are quite profound when different doses of antibiotics are used. In contrast to high-dose antibiotic treatment, low-dose antibiotic exposure during early life induces long-term metabolic impacts including the enhancement of high-fat diet-induced body weight and fat mass, and hepatic steatosis in C57BL/6J mice [35]. 

Recently, a study has indicated that the alteration in gut microbiota produced by vancomycin and bacitracin improves glucose tolerance, hyperinsulinemia and insulin resistance in high-fat diet fed mice in which the abundance of *Firmicutes* and *Bacteroidetes* bacteria was significantly reduced. Further investigations have demonstrated that the improvement in insulin sensitivity is due to the increased secretion of glucagon-like peptide 1 (GLP-1) in antibiotic-treated mice. The increased secretion of GLP-1 results from an elevated taurocholic acid (TCA) level that is associated with the depletion of *Firmicutes* and *Bacteroidetes* bacteria [36]. Therefore, the alteration of gut microbiota is a contributing factor in the development of insulin resistance, which highlights the potential for improving insulin sensitivity by modulating gut microbiota. 

### 2.3. Gut Microbiota and Choline Metabolism

Choline is an important component of cell and mitochondrial membranes, and acetylcholine, which plays critical roles in various physiological processes such as lipid metabolism, signal transduction of second messengers, enterohepatic circulation of bile acid and cholesterol metabolism [37]. Accordingly, choline deficiency usually causes fatty liver formation [38,39]. PEMT and MAT1 are two critical genes in choline metabolism by catalyzing phosphatidylethanolamine into phosphatidylcholine and the formation of endogenous choline. The deletion of the PEMT or MAT1 genes results in the development of fatty liver disease in animals [40,41]. In addition to dietary and genetic factors, the endogenous level of choline is also influenced by gut microbiota [42,43]. 

Melanie *et al.* [44] investigated the relationship between low-choline diet-induced fatty liver and the change of gut microbiota in 15 healthy women who accepted a two-month diet intervention with different levels of choline, including 10 days of normal diet (contain 550 mg of choline, 50 mg betaine/70 kg body weight), 42 days of low-choline diet (<50 mg choline, 6 mg betaine/70 kg body weight) and 10 days of high-choline diet (850 mg choline/70 kg body weight). It was shown that short-term intervention with identical diets did not change the overall structure of original gut microbiota among individuals. However, the composition of gut microbiota before the low-choline diet intervention is correlated with the formation of fatty liver disease, in which the differences in abundance of *Gammaproteobacteria* and *Erysipelotrichi* bacteria are associated with the content of fat in hepatocytes. Given the well-established relationship between single nucleotide polymorphisms of the PEMT gene and susceptibility to low-choline diet-induced fatty liver, the authors further evaluated the predictive capability of fatty liver development among individuals with an abundance of *Gammaproteobacteria* and *Erysipelotrichi* bacteria, and the PEMT gene polymorphism. They found that the combination of bacteria and PEMT gene polymorphism information provided good prediction towards low-choline diet- induced fatty liver formation [44]. This study highlights the complicated impact on fatty liver development by intricate interactions between host genetics, gut microbiota and dietary intervention. The elucidation of the gut microbial modulation of choline metabolism provides new insights into the pathophysiology of NAFLD. 

### 2.4. Gut Microbiota and Bile Acid Metabolism

Bile acids are synthesized from cholesterol by various enzymes in hepatocytes, which are stored in the gall bladder, and secreted into the duodenum during a meal [45]. Over 95% of bile acids in the small intestine are reabsorbed by intestinal cells through the ileal bile acid transporter (IBAT/ASBT) or passive diffusion, and then recycled via the portal vein into the liver [45]. In addition to promoting the absorption of fat, cholesterol and fat-soluble vitamins in the intestinal tract, bile acids also function as signaling molecules that modulate a variety of physiological processes. These include the homeostasis of bile acid metabolism itself, and metabolism of lipoprotein and glucose by regulating nuclear receptors such as the farnesoid X receptor (FXR), the G protein-coupled receptor TGR5 and so on [46,47,48]. Therefore, bile acids and their metabolites play crucial roles in maintaining the homeostasis of triglyceride, cholesterol and glucose in the liver. The dysregulation of bile acid homeostasis and its regulated signaling pathways contributes to the occurrence of NAFLD [45]. 

Bile acids have potent antimicrobial activity [49], while the composition of gut microbiota also produces a strong impact on bile acid metabolism [50]. Cholic acid (CA) and chenodeoxycholic acid (CDCA) are mainly produced in the liver of humans, but CA and β-muricholic acid (βMCA) in mice [51]. The synthesized bile acids are conjugated with glycine or taurine in hepatocytes and secreted into the intestinal tract via the duodenum, which are then largely deconjugated and metabolized into different forms of bile acids by gut microbiota [50]. The high-fat diet-induced metabolic changes including bile acid alterations are associated with the remodeling of gut microbiota [52,53], and the dysregulated bile acid pool alters the composition of the gut microbiota [52], which in turn contributes to NAFLD formation by influencing lipid and energy metabolism. 

FXR is a central sensor and modulator for bile acid and lipid metabolism, which is mainly expressed in the liver and intestine [54,55]. Gut microbiota can modulate the activity of FXR in the intestine that affects lipid metabolism in the liver [56,57,58]. In a recent study [59], researchers determined the relationship between gut microbiota and the formation of NAFLD in a high-fat diet fed murine model by administering antibiotics to disturb the composition of the gut microbiota. They found that antibiotic treatment attenuated the development of NAFLD accompanied with alterations in the bile acid composition, and inhibition of intestinal FXR signaling. Moreover, mice with intestine-specific *Fxr* disruption had fewer accumulations of hepatic triglyceride in response to a high-fat diet compared to control mice. Further investigations revealed that the resistance to a high-fat diet-induced NAFLD in intestine-specific *Fxr* knockout mice was mainly due to a reduction of circulating ceramide, which results in the downregulation of hepatic SREBP1C and suppression of *de novo* lipogenesis. The study demonstrated the intestinal FXR/ceramide axis could be a novel target for NAFLD therapy, in which the alteration of gut microbiota is critically involved. However, it is still controversial about the exact role of FXR in NAFLD formation. Whole-body deletion of FXR results in significant accumulation of hepatic and circulating triglyceride, as well as VLDL, LDL and HDL [60,61], whereas activation of FXR with the agonist GW4064 increased high-fat diet-induced NAFLD [62]. Some studies indicate that an activated hepatic FXR/SHP pathway suppresses hepatic lipogenesis and SREBP1C [63,64,65]. On the contrary, a natural FXR antagonist, guggulsterone, has been found to decrease hepatic cholesterol and plasma triglyceride [66,67]. Although the function of FXR in hepatic lipid metabolism remains to be clarified, the gut microbial modulation by FXR highlights the profound effects of gut microbiota on lipid signaling pathways. 

### 2.5. Gut Microbiota-Targeted Therapy of NAFLD

The increasing evidence for the functions of gut microbiota in the development of NAFLD provides an important rationale for developing a gut microbiota-targeted strategy to prevent or treat NAFLD. The most commonly used ways for interfering with gut microbiota include probiotic, prebiotic and synbiotic supplements, or even antibiotic treatment. Bacteria of *Lactobacillus*, *Streptococcus* and *Bifidobacterium* are frequently used for their beneficial effects on metabolic diseases [68,69,70]. Cai *et al.* [71] investigated the effects of *Lactobacillus* and *Bifidobacterium* bacteria on high-fat diet-induced NAFLD mice; they found that the hepatic TG content was significantly reduced in the *Bifidobacterium* supplemented group, which was better than *Lactobacillus* in reducing liver lipids. Shen *et al.* [72] observed the protective effects of probiotics on three different bacteria, *Lactobacillus paracasei* (*Lactobacillus paracasei* CNCM I-4270), *Lactobacillus rhamnosus* (*L. rhamnosus* I-3690) and *Bifidobacterium lactis* (*Bifidobacterium animalis* subsp. *lactis* I-2494) in high-fat diet-induced metabolic syndrome mice. The results showed that all these probiotics could effectively reduce the extent of liver steatosis, but show variable improvement in reducing inflammatory reactions, which suggests that the observed probiotics may function through different mechanisms. Fontana *et al.* [73] observed protective effects against steatosis of *Lactobacillus paracasei* (CNCM I-4034), *Bifidobacterium breve* (CNCM I-4035), *Lactobacillus rhamnosus* (CNCM I-4036), as well as the combination of *L. paracasei* with *B. breve* on Zucker obese rats. They reported that these probiotics could reduce hepatic steatosis, except for *Lactobacillus paracasei*. Moreover, all these probiotics can reduce the plasma inflammatory cytokines (such as LPS, TNF-α, IL-6) to different extents, suggesting that the beneficial effects of probiotics against liver steatosis may be associated with a reduction in inflammatory reactions.

In the clinic, probiotic VSL#3 or in combination with other bacteria has been trialed in NAFLD patients, who showed improvements in serum transaminase, TNF-α and oxidative stress after 2–3 months of therapy [74,75]. In addition to the direct metabolic modulation, probiotics can also prevent adherence of detrimental bacteria to the intestinal mucosa [76,77], produce antimicrobial peptides [78], reduce inflammation and enhance the immune functions of the host [79], which are all beneficial effects in the treatment of chronic liver injury.

Prebiotic is a collection of non-degradable food ingredients including fructan, oligosaccharides, lactulose, resistant starch, and so on. These food ingredients cannot be directly absorbed and used by humans. They must be transformed into absorbable metabolites by intestinal bacteria such as SCFAs (acetic acid, propionic acid, butyric acid). Meanwhile, prebiotic can benefit host metabolism by stimulating the growth of beneficial bacteria such as *Lactobacilli* and *Bifidobacteria* [80]. The microbial-derived SCFAs are not only involved in affecting the host energy harvest [81], but also act as signaling molecules to activate the corresponding receptors on targeted organs such as G protein-coupled receptors [82], free fatty acids receptor 2/3, *etc.* [83,84]. 

In addition, prebiotics can regulate the appetite of the host and energy metabolism by altering the release of various gastrointestinal peptides [85,86]. In obese animals, the addition of 10% fructan to the diet can increase the release of anorexia gastrointestinal peptide (PYY, GLP-1), but reduce the release of ghrelin, which stimulates appetite [87]. The satiety effect mediated by prebiotics has been further confirmed in a GLP-1 receptor defective animal model. Prebiotics can improve the intestinal endocannabinoids system, lower the permeability of the intestinal wall, attenuate metabolic endotoxemia, and eventually reduce the accumulation of body fat, and prevent obesity and the development of NAFLD [88].

Symbiotics are a combination of a probiotics and prebiotics. In NASH patients, researchers observed the effects on serum ALT and AST of continuous treatment with a combination of *Bifidobacterium* (*Bifidobacterium longum*) and fructo-oligosaccharide for six months. The results showed that the serum AST level of patients given symbiotic treatment was significantly lower than in the control group [89], which suggests the potential benefit of symbiotics for the treatment of NASH. However, similar beneficial effects were not observed in another randomized controlled study, in which the symbiotic of *Lactobacillus*, *Bifidobacterium* and fructo-oligosaccharide was used [90].

**Figure 1:**
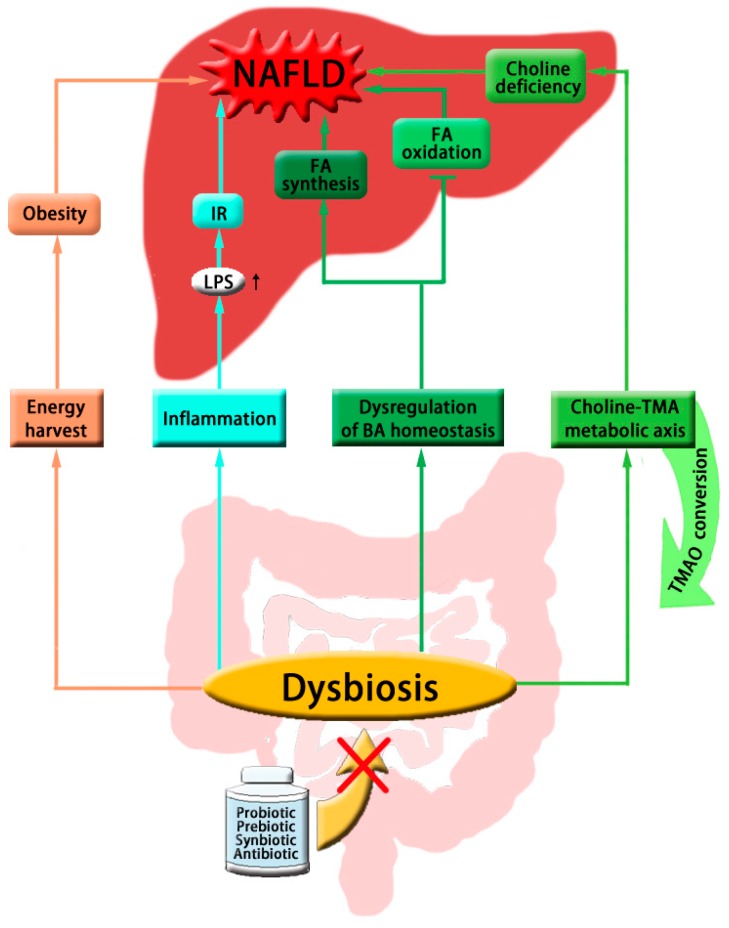
Schematic summary of the gastrointestinal microbial contribution to the development of NAFLD. The colored arrows represent stimulated effect, as well as the inhibitory effect shown with cross line. The arrows with identical color means same pathway involved, and the black arrow indicates increased secretion of LPS. Abbreviations: NAFLD: nonalcoholic fatty liver disease; FA: fatty acid; IR: insulin resistance; LPS: lipopolysaccharide; BA: bile acid; TMA: trimethylamine; TMAO: trimethylamine-*N*-oxide.

Although exciting evidence of probiotics, prebiotics or symbiotics has been acquired, some of the results have discrepancies among different studies, as well as between experimental and clinical trials. Given the huge numbers of bacteria and the bacteria composition in the intestinal tract, there are clearly many factors that may lead to the inconsistency in therapeutic outcomes of certain probiotics, such as differences in bacteria preparation, host genetics, diet and lifestyle. At this moment, it is inclusive on the exact outcomes of gut microbiota-targeted therapies, which needs further experimental and larger-scale clinical investigations [58]. The contributions made by gut microbiota to NAFLD are summarized in Figure 1.

## 3. Application of Metabolomics in Characterizing the Role of Gut Microbiota in NAFLD

### 3.1. Gut Microbial Metabotypes 

The co-metabolism of gut microbiota in a host means that a large number of microbial metabolites will be excreted in blood, urine or the feces [91]. These metabolites can be indicators of the metabolic status of specific diseases or therapy, and some of the microbial metabolites possess important biological functions through their direct modulation on enzymatic activity in the host, or regulation of host signaling pathways [1]. 

Metabolomics is one of the system biological approaches that can capture the fluctuation of entire or specific types of endogenous metabolites by using nuclear magnetic resonance (NMR) spectroscopy or mass spectrometry-based metabolomic platforms, including gas chromatography-mass spectrometry (GC-MS), liquid chromatography-mass spectrometry (LC-MS) and subsequent data analysis. The choice and characteristics of each platform for a metabolomic study have been well discussed in a previous review [92]. The microbial-specific metabotypes (metabolic phenotypes) are usually obtained with the metabolomic approach through comparisons between conventional and antibiotic-treated animals. In one of our studies [93], we performed a dynamic metabolomic profile on urinary and fecal metabolites using combined GC-MS and LC-MS approaches in rats with or without antibiotic exposure. The antibiotic exposure resulted in time-dependent “fingerprints” of the urine and feces in which over 200 microbial-related metabolites were identified, such as SCFAs, metabolites of tryptophan, tyrosine and indole metabolism. This study highlights the potential of a metabolomic approach in visualizing the gut microbial metabotypes that are related to the endpoints of a “functional metagenome” (functional members of the microbiome that influences host metabolism and health) [91]. The evidence for gut microbial metabotypes has gradually accumulated in germ-free animals using metabolomics. A distinct microbial metabotype is observed in various tissues in germ-free mice compared to their conventional counterparts [94], indicating the systemic metabolic impacts of gut microbiota, in addition to the local intestinal effects. Accordingly, the metabolomic approach is potent for characterizing the involvement of gut microbiota in host metabolism. 

### 3.2. Gut Microbial Metabotypes and NAFLD 

Given the capability of metabolomics in characterizing the gut microbial metabotypes, the application of metabolomics in NAFLD provides an important approach for uncovering the gut microbiota-involved mechanisms in NAFLD. A strain of 129S6 mice is known to be susceptible to dietary-induced impaired glucose homeostasis and NAFLD. Nicholson *et al.* [95] performed metabotyping on plasma and urine samples of 129S6 mice with ^1^H-NMR spectroscopy and revealed that the genetic predisposition of 129S6 mice to impaired glucose tolerance and NAFLD was due to the gut microbial disruption of choline metabolism. The gut microbiota in 129S6 mice converts choline into methylamines such as dimethylamine (DMA), trimethylamine (TMA), and trimethylamine-*N*-oxide (TMAO) in a high-fat diet leading to a reduction in choline bioavailability that mimics the effects of a choline-deficient diet. In a high-fat-diet-induced NAFLD model, metabolomic approach has also been used to evaluate the holistically protective activity of garlic acid, which not only restores the high-fat diet-induced metabolic alteration including lipids, glucose, amino acids and choline metabolism, but also impacts on gut microbiota-involved metabolism such as hippurate, DMA and TMA [96], suggesting that metabolomics has the potential for uncovering the gut microbial involvement in NAFLD therapy by natural medicine.

Although metabolomics has been applied in NAFLD studies, the characterization of gut microbial metabotypes in NAFLD largely relies on the identification of gut microbial metabolites. Currently, there are several types of gut microbial metabolites that are well confirmed to have important biological functions, or as biomarkers for gut microbial involvement in metabolic diseases. These gut microbial metabolites include SCFAs, bile acids and choline metabolites (DMA, TMA, TMAO, dimethylglycine, betaine), indole and phenolic derivatives, and lipids [1] (summarized in Table 1). The variation of SCFAs is commonly observed in obesity [19], insulin resistance [97], type 2 diabetes [98] and NAFLD [99] because of their contribution to obesity development. Kalhan *et al.* [100] performed a plasma metabolomic profile on a group of NAFLD subjects with either hepatic steatosis or steatohepatitis. They found that the plasma concentrations of GCA, TCA and GCDCA in steatohepatitis patients, whereas only TCA in steatosis patients, were significantly higher than in control subjects. The fluctuation of these bile acids identified with a metabolomic profile could potentially be used as biomarkers for evaluating the status of NAFLD and the therapeutic outcome. 

## 4. Conclusions

The intricate relationship between gut microbiota and NAFLD is now well recognized due to the advancement in analytical platforms such as metabolomics, metagenomics and other techniques. However, it is a great challenge to elucidate the exact roles of gut microbiota in the development of NAFLD. Since most of the intestinal bacteria are unculturable, the cultivation-independent metagenomics revolutionizes the study of microbial involvement in many diseases, but the relatively high cost of whole bacteria DNA sequencing limits its application in small laboratories. Alternatively, the sequencing of the 16S rRNA gene is widely used for phylogenetic studies of bacteria on the basis of its hypervariable regions. The sequencing of hypervariable regions in the 16S rRNA gene is a rapid and cheap way to identify bacteria, especially at the level of the genus or family. 

In addition, metabolomics is a complimentary approach for uncovering the involvement of gut microbiota in disease due to the established correlation of some microbial-derived metabolites with certain species of bacteria. For example, SCFAs such as acetate, propionate, butyrate, isobutyrate are produced by species of *Eubacterium*, *Roseburia*, *Faecalibacterium*, *Coprococcus*, and *Clostridial* clusters IV and XIVa in the *Firmicutes* family that have various important functions in host energy metabolism [1]. Nevertheless, only some components of the gastrointestinal microbial metabolites have been connected with corresponding bacteria at species or genus level to date. More research is needed to bridge the correlation between gut microbial metabolites and bacteria, as well as the integration of different omic approaches.

## Figures and Tables

**Table 1:** Microbial metabolites and their implicated diseases.

Type of Metabolites	Representative Microbial Metabolites	Biological Functions	Implicated Diseases	Ref.
Short chain fatty acids	Butyrate, acetate, propionate	Anti-inflammatory, induce apoptosis and suppress histone deacetylase; provide energy for gastrointestinal tract epithelial cells; inhibit fatty acid and cholesterol synthesis	Colorectal cancer, diabetes mellitus, insulin resistance and lipid disorders	[21,101,102,103,104]
Bile acids	UDCA	Suppresses bile-induced pancreatic ductal injury by reducing apoptosis and mitochondrial damage; inhibits DCA-induced apoptosis and its signaling pathway in oesophageal cancer cells	Cancer, gallstone disease, primary biliary cirrhosis	[105,106]
	DCA	Involved in senescence-associated secretory phenotype that facilitates hepatocellular carcinoma development in mice; contributes to the development of colorectal cancer by decreasing miR-199a-5p expression, or upregulating invasive and angiogenic potentials of human colon cancer cells	Hepatocellular carcinoma and colorectal cancer	[107,108,109]
Choline	TMAO, TMA, DMA	Elevated plasma levels of TMAO positively correlated with increased risk of major adverse cardiovascular events and colorectal cancer; impairs reverse cholesterol transportation; contributes to renal fibrosis and dysfunction in animals	Cardiovascular diseases, cancers, kidney disease	[110,111,112,113]
	betaine	Regulates the LXRα/PPARα pathway and alleviates ER stress; predictor of cardiovascular outcomes	NAFLD, diabetes	[114,115]
Phenyl derivatives	hippuric acid, hydroxyhippuric acid, benzoic acid, *p*-cresol, phenylacetylglycine, phenylacetylglutamine	Biomarkers for the involvement and variation of gut microbiota in diseases; detoxification of xenobiotics	Obesity, metabolic diseases, cancers, pancreatitis	[116,117,118,119,120]
Indole derivatives	*N*-acetyltryptophan, indoleacetate	Indicates the defect in tryptophan metabolism; uremic toxins;	Chronic renal failure	[121,122]
	serotonin, melatonin	Involved in brain-gut axis function; reduce inflammatory infiltration, regulate adipokine secretion and energy expenditure, improve insulin sensitivity	Anxiety, depression, gastrointestinal dysfunction, obesity, metabolic syndrome	[123,124,125,126,127]
Polyamines	putrescine, spermidine, spermine	Stabilize DNA, RNA and protein molecules, regulate cell proliferation and differentiation; biomarkers of human diseases	Cancer, stroke, renal failure, obesity and diabetes	[128,129,130]
Lipids	LPS	Impairs intestinal permeability, induces systemic inflammation and insulin resistance	obesity, diabetes	[131,132]
	phosphatidylcholines	Altered in patients with Alzheimer’s disease or esophageal squamous cell carcinoma	Alzheimer’s disease, patients with esophageal squamous cell carcinoma	[133,134]
